# Comparative Analysis of *Salmonella enterica* subsp. *enterica* Serovar Thompson Isolates associated with Outbreaks Using PFGE and wgMLST

**DOI:** 10.4014/jmb.2210.10010

**Published:** 2022-11-15

**Authors:** Youngho Koh, Yunyoung Bae, Min-Jung Lee, Yu-Si Lee, Dong-Hyun Kang, Soon Han Kim

**Affiliations:** 1 Food Microbiology Division, National Institute of Food and Drug Safety Evaluation, Ministry of Food and Drug Safety, Cheongju 28159, Republic of Korea; 2 Department of Food and Animal Biotechnology, Department of Agricultural Biotechnology, Center for Food and Bioconvergence, Research Institute for Agricultural and Life Science, Seoul National University, Seoul 08826, Republic of Korea

**Keywords:** *Salmonella enterica* serovar Thompson, foodborne outbreak, chocolate cake, pulsed-field gel electrophoresis, whole-genome multi-locus sequence typing

## Abstract

The strains associated with foodborne *Salmonella enterica* Thompson outbreaks in Korea have not been identified. Therefore, we characterized *S*. Thompson strains isolated from chocolate cakes linked to foodborne outbreaks in Korea. A total of 56 strains were isolated from preserved cake products, products in the supply chain distribution, the manufacturer’s apparatus, and egg white liquid products used for cream preparation. Subsequently, serological typing, pathogenic gene-targeted polymerase chain reaction (PCR), pulsed-field gel electrophoresis (PFGE), and whole-genome multi-locus sequence typing (wgMLST) were performed to characterize these isolates. The antigen formula of all isolates was 7:k:1,5, namely *Salmonella enterica* subsp. *enterica* Serovar Thompson. All 56 isolates harbored *invA*, *his*, *hin*, and *stn*, and were negative for *sefA* and *spvC* based on gene-targeted PCR analyses. Based on PFGE results, these isolates were classified into one group based on the same SP6X01.011 pattern with 100% similarity. We selected 19 strains based on the region and sample type, which were subjected to wgMLST. Although the examined strains showed 100% similarity, they were classified into seven clusters based on allelic differences. According to our findings, the cause of these outbreaks was chocolate cake manufactured with egg white liquid contaminated with the same *Salmonella* Thompson. Additionally, comparative analysis of wgMLST on domestic isolates of *S*. Thompson from the three outbreaks showed genetic similarities of over 99.6%. Based on the results, the PFGE and wgMLST combination can provide highly resolved phylogeny and reliable evidence during *Salmonella* outbreak investigations.

## Introduction

In Korea, non-typhoidal *Salmonella* represents one of the most frequent causes of foodborne disease outbreaks, following norovirus and pathogenic *Escherichia coli*. Food poisoning caused by *Salmonella* spp. is a global health threat, with 80.3 million cases reported annually [1]. Among the different species of *Salmonella*, *Salmonella enterica* has long been subclassified into serovars based on differential antibody reactions [2]. The use of specific antibodies that can identify distinct cell-surface antigens within lipopolysaccharides and flagella has led to the identification of over 2,500 serovars that differ in their antigenic formulas [3].

*S*. Enteritidis and *S*. Typhimurium that cause human salmonellosis are major serotypes found globally. Recently, mass infection cases caused by major serotypes as well as other serotypes have been reported. According to data from the National Institute of Health, the Korea Disease Control and Prevention Agency, infections related to the serotype of *Salmonella* in Korea show a diverse isolation trend every year, and in 2015, major serotypes isolated were in the following order: *Salmonella* Enteritidis, *Salmonella* I 4,[5],12:i:-, S. Bareilly, *S*. Typhimurium, and *S*. Montevideo [4, 5].

Due to the foodborne outbreak caused by *S*. Thompson in 2018, which is addressed in the present study, a total of 3,516 patients infected with *Salmonella* spp. were identified in 2018, including 2,975 patients from 12 cities and provinces across the country, and a prevalence of 3.6 times the annual average was recorded according to the statistical report of the Ministry of Food and Drug Safety (MFDS) [6].

In September 2018, 60 school meal services, including those in kindergartens, reported foodborne outbreaks [7], and 56 isolates were collected from the traceback investigation by regional public health centers and the MFDS. This outbreak involved the second-highest number of cases to occur in a school outbreak nationwide, following the norovirus school outbreak (over 3,000) in 2006 and the highest number of cases in a *Salmonella* outbreak in Korea.

When foodborne outbreaks occur, one of the most important laboratory analyses involves the determination of correlation among the strains. For example, if a strain isolated from a specimen of the patient matches exactly with that isolated from food samples, then the strain is confirmed as the cause of the outbreak. To investigate whether the source and isolates of these outbreaks were identical or independent and further matched with the isolates obtained from specimens, we analyzed the phenotypic and genotypic characteristics of 56 strains in this study. Various methods are used to determine the similarities between strains, and the pulsed-field gel electrophoresis (PFGE) analysis method has long been used as the gold standard worldwide. Recently, various whole-genome sequencing analysis methods with relatively higher accuracy and precision have been researched with the development and generalization of next-generation sequencing technology. One of them is whole-genome multi-locus sequence typing (wgMLST), which is used as a standard method in PulseNet International [8]. In the present study, wgMLST was used for the analysis of correlation with outbreak strains, leading to more accurate results with high resolution, and we described the laboratory procedure for confirming the sources of outbreak via characterization analysis.

## Materials and Methods

### *S*. Thompson Isolation

*S*. Thompson strains were isolated according to the methods of the Korean Food Code and Guidelines for Laboratory Analysis of Foodborne Outbreaks for food and environmental samples collected during the traceback investigation in six cities (Seoul, Busan, Daegu, Gwangju, Ulsan, and Jeju) and four provinces (Chungbuk, Gyeongbuk, Gyeongnam, and Jeonbuk) in South Korea. A total of 56 strains were isolated from preserved cake products, products in the supply chain distribution, the manufacturer’s apparatus, and egg white liquid products used for cream preparation. A 25 g sample and 225 ml of buffered peptone water (Oxoid, United Kingdom) were mixed thoroughly and enriched in an incubator at 37°C for approximately 24 h. The enriched culture solution was added to two enrichment media, 1 ml of culture was added to 10 ml of tetrathionate medium (BioMérieux, Spain) and 0.1 ml was added to 10 ml of Rappaport-Vassiliadis medium (Oxoid), which underwent secondary enrichment at 37°C in tetrathionate medium and 42°C in Rappaport-Vassiliadis medium for 20–24 h. The secondary enrichment culture solution was smeared on selective media including XLD agar (Oxoid) and brilliant green sulfa agar (Remel, United Kingdom) and cultured at 37°C for 18–24 h, and then a typical colony was selected and subcultured in the nutrient medium following identification using Vitek MS (BioMérieux Inc., France).

### Serology Testing

Serological tests were conducted in accordance with the method provided by the MFDS [4] to verify serotypes of isolated strains. Antisera (BD, Difco) with somatic (O) antigen (A, B, C, D, E, and Vi) and flagellar (H) antigens (a, b, c, d, e, h, i, k, r, y, and z) were used to perform slide and tube agglutination tests to identify the serotypes.

### Pathogenic gene analysis using polymerase chain reaction (PCR)

Virulence genes were screened for using PCR for 56 isolates. To extract DNA from pure strains, a single colony was taken and DNA was extracted using automated equipment (EZ1 Advance XL; Qiagen, United Kingdom) according to the manufacturer’s specifications and then used as the DNA template. The target genes were *invA*, *his*, *Salmonella* enterotoxin (*stn*), *sefA*, *spvC*, and *hin* according to the following references: For *his*, *invA*, and *stn* gene detection in *Salmonella* spp., 5 μl of the template DNA was added to the mixture using a detection kit Powercheck *Salmonella* spp. (Kogenbiotech Co., Ltd., Korea) according to the method proposed by the manufacturer and made up to a total volume of 20 μl, using which real-time PCR (7500 Fast real-time PCR; Applied Biosystems, USA) was performed. For *spvC*, *sefA*, and *hin* gene detection, real-time PCR and conventional PCR were performed according to the methods of Bugarel *et al*. [9], Seo *et al*. [10], and Kim *et al*. [11]; the primer/probe and PCR conditions used are listed in Table 1.

For *spvC* and *sefA* detection, 5 μl of the extracted DNA, 1 μl and 1.5 μl of forward and reverse primers (10 pmol/μl), respectively, 0.5 μl of probe (10 pmol/μl), and PCR master mix (Kogenbiotech) were used to make a final volume of 20 μl, and real-time PCR (7500 Fast real-time PCR, Applied Biosystems) was performed.

For *hin* gene detection, 5 μl of the extracted DNA, 1 μl of forward and reverse primers (10 pmol/μl) each, and PCR master mix (Bioneer, Korea) were made up to a final volume of 20 μL and real-time PCR (C1000 Touch Thermal Cycler; Bio-Rad, USA) was performed. Based on the PCR products, a specific band was verified via electrophoresis on a 2% agarose gel.

### PFGE

PFGE analysis of *Salmonella* spp. was performed in accordance with the PFGE Standard Testing published by the MFDS. Cultures of pure strains were added to cell suspension TE buffer (100 mM Tris and 100 mM EDTA, pH 8.0), and the concentration was adjusted to an optical density of 0.8–1.0 measured at 610 nm using a spectrophotometer. Subsequently, 200 μl of 1.2% Seakem Gold agarose was added to the strain suspension, mixed gently, and immediately solidified in a plug mold. The solidified plug was transferred to 1.5 ml cell lysis buffer (50 mM Tris, 50 mM EDTA, pH 8.0; 1% sodium lauroyl sarcosine) to which 50 μl of proteinase K was added. After incubation in a 55°C shaking water bath for 1.5 to 2 h, the plug was washed five times with a plug wash TE buffer (10 mM Tris, 1 mM EDTA, pH 8.0) for 20 min. A 1-millimeter thick slice was cut from the washed plug and incubated at 37°C for 2 h with 40 U/μl XbaI (Roche Diagnostics, Switzerland). Electrophoresis was performed using the plug gel treated with XbaI at 14°C for 18 h under the conditions: initial time 2.16 s, final time 63.8 s, gradient 6 V/cm; and an angle of 120°. *S. enterica* serotype Braenderup BAA-664 standards were used as the size marker, and testing was performed in the same manner for the isolated strains. After electrophoresis, the gel was placed into SYBR gold stain (Invitrogen, USA) and dyed for 30 min, after which UV was used for identification. Identified pictures were analyzed using the program, BioNumerics (Applied Maths, Belgium).

### wgMLST Analysis

For whole-genome sequencing, genomic DNA was extracted from *S*. Thompson using the MagListo 5 M Genomic DNA Extraction Kit (Bioneer). Sequencing libraries were prepared using the Nextera DNA Flex Kit (Illumina, USA), and paired-end reads were generated using the MiSeq Reagent Kit v2 (Illumina) using the MiSeq sequencing platform (Illumina) [12]. After the entire genome sequencing process, allele calls with genome assembly were determined for wgMLST analysis. BioNumerics version 7.6 (Applied Maths, Belgium) suite of software applications was used to perform wgMLST analysis [13]. Cluster analysis of categorical values of allelic numbers for wgMLST was used to construct phylogenetic networks using the unweighted pair group method with arithmetic mean algorithms. Among the 56 strains analyzed in this study, wgMLST analysis was performed for 19 strains, excluding strains with the same sample characteristics and isolation area. The 19 strains analyzed included two strains from two cakes (MFDS1011643 and MFDS1011640), two strains from egg white liquid products (MFDS1011653 and MFDS1011655), one strain from the manufacturing apparatus: whipper (MFDS1011714), and 14 strains from preserved food samples (MFDS1011687, 1011692, 1011683, 1011679, 1011694, 1011682, 1011686, 1011691, 1011693, 1011704, 1011705, 1011712, 1011730, and 1011702).

### Correlation Analysis among *S*. Thompson Isolates Related to Foodborne Outbreaks

The correlation between the strains isolated from foodborne diseases in 2018 and *S*. Thompson isolates from the previous two outbreaks in Korea was analyzed using the PFGE method. The two strains from outbreaks in 2014 and 2015, which showed highly similar PFGE patterns with those of the strains isolated from egg white liquid samples, were additionally analyzed using the wgMLST analysis method.

## Results

Isolation and Identification of *Salmonella* spp.

*S*. Thompson strains were isolated from the samples collected during the outbreak investigation process in six cities, including Seoul, Busan, Daegu, Gwangju, Ulsan, and Jeju, and four provinces, including Chungbuk, Gyeongbuk, Gyeongnam, and Jeonbuk. The list of 56 *S*. Thompson strains isolated from a total of 56 samples, including three cakes produced by a manufacturer, one used manufacturing apparatus, two egg white liquid products, and 50 preserved foods, is shown in Table 2.

### Confirmation of Serotype

Serological testing of 56 *Salmonella* isolates revealed that the O-antigen group contained a bacterial strain that belonged to serotype C. *In silico* serotyping predicted an antigenic profile of 7:k:1,5. All strains showed O:7 agglutination, flagella H-antigen phase 1 on k, and phase 2 on 1 and 5. Finally, 56 strains with identical serotypes were confirmed to be *S*. Thompson according to the Kauffman-White scheme [14].

### PCR Analysis of Pathogenic Genes

PCR analysis of the strains isolated from the samples revealed that all bacterial strains harbored *invA*, *his*, *hin*, and *stn* and were negative for *sefA* and *spvC* (Table 3). The following genes related to *Salmonella* spp. were detected: *inv* is related to adhesion to and invasion into epithelial cells [15], *his* is involved in regulating histidine transport [16]; *sefA* encodes fimbria to specifically detect *S*. Enteritidis [17], *spv* causes cytotoxicity upon moving into the host cell, *stn* causing diarrhea when *Salmonella* spp. invade the intestines, and *hin* expresses flagella corresponding to two flagellar antigen phases 1 and 2. PCR is used to specifically detect *Salmonella* by identifying the related genes [18]. In this study, all *S*. Thompson isolates had the following genes: *invA*, *hin*, *stn*, and *his*. PCR analysis of *hin* identified a *hin*-specific product of 572 bp in all *Salmonella* strains.

### PFGE Patterns of *S*. Thompson

**Fig. 1:**
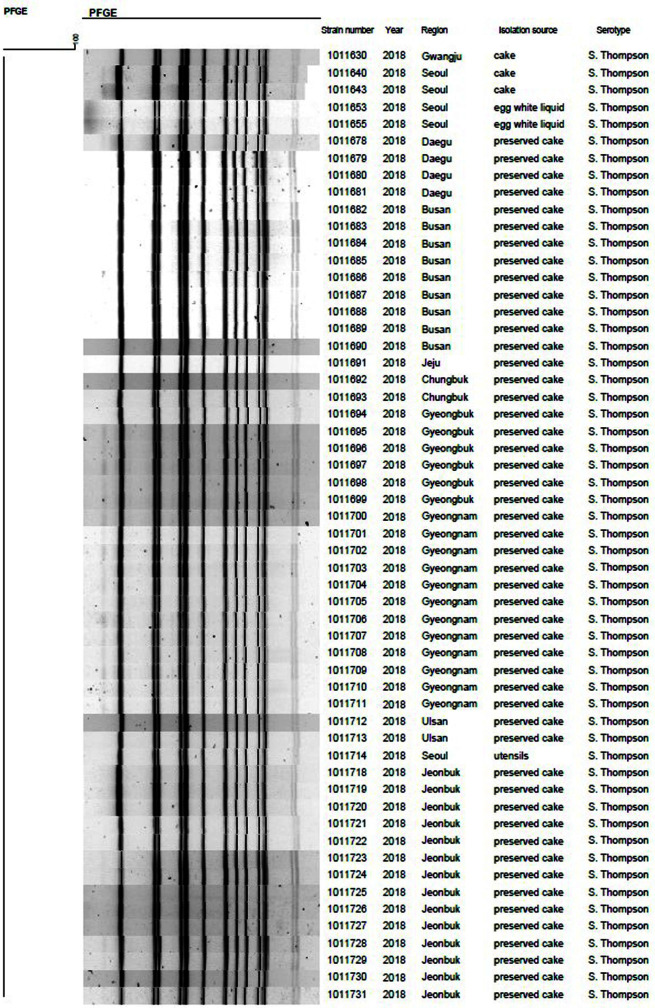
Similarity of 56 *Salmonella* Thompson isolates determined via pulsed-field gel electrophoresis analysis using XbaI.

Based on PFGE results, the 56 *S*. Thompson isolates were classified into one cluster with the same pattern as shown in Fig. 1, which showed 100% identical genetic similarity.

### wgMLST

Nineteen strains were analyzed which comprised 14 strains isolated from the preserved cakes selected by region and sample type, including one strain at a time, two strains from egg white liquid products, two strains from the cake products in the supply chain distribution, and one strain from the manufacturer’s apparatus, a whipper. These strains showed the same genotype in PFGE and 100% similarity in wgMLST; however, they were classified into seven clusters. The clusters were classified based on the number of allelic differences, which were small based on 2 to 3 alleles. Therefore, in terms of similarity (%), the difference was trivial, leading to the conclusion that they have the same similarity level.

### Correlation among *S*. Thompson Strains Isolated in Korea

To determine the correlation between isolated *Salmonella* and foodborne outbreaks, three strains associated with three foodborne outbreaks were analyzed using PFGE and wgMLST. In addition to one strain isolated from the outbreak in 2018, two strains isolated from a restaurant in Jeonnam-do (2014) and a restaurant in Jeonbuk-do (2015) were analyzed. The strain isolated from an ingredient of gimbab (crab stick) in 2014 and from rolled egg omelets provided by a restaurant in 2015 belonged to the strain numbers MFDS1004024 and MFDS100681, respectively. The three strains had the same genotype, sequence type, and characteristics, as summarized in Table 4.

**Fig. 3:**
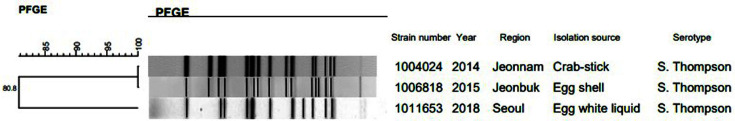
Pulsed-field gel electrophoresis patterns of *Salmonella* Thompson isolates obtained from three *Salmonella* outbreaks in Korea.

The results of PFGE analysis of the three strains showed that the two strains (MFDS1004024 and MFDS1006818) isolated from different outbreaks in 2014 and 2015, respectively were 100% identical as shown in Fig. 3, and showed 80% similarity with MFDS1011653, a strain from 2018, indicating a relatively lower level of similarity.

**Fig. 4:**
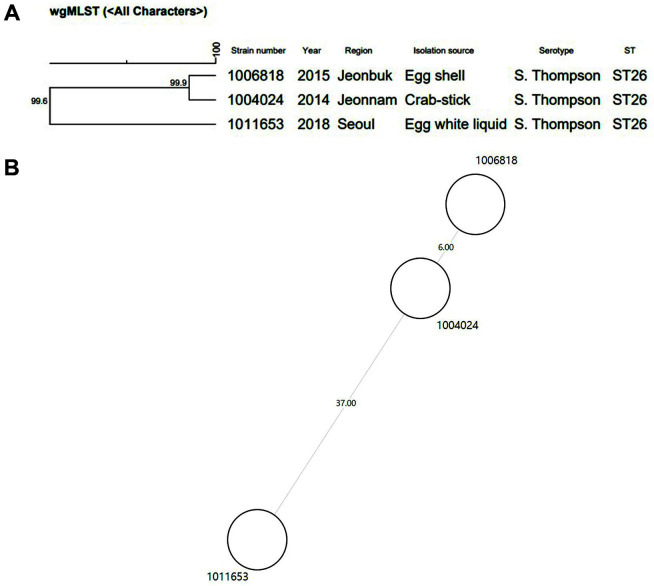
A) Similarity between domestic *Salmonella* Thompson isolates determined via wgMLST analysis. The isolates of MFDS1004024 and MFDS1006818 showed 99.9% similarity. MFDS1011653 showed 99.6% similarity with MFDS1004024 and MFDS1006818. **B**. Minimum spanning tree of three isolates showed 37 allelic differences between MFDS1004024 and MFDS1011653 and 6 allelic differences between MFDS1004024 and MFDS1006818. MST was generated in BioNumerics 7.6 using wgMLST data for isolates sequenced in this study. wgMLST, whole-genome multi-locus sequence typing.

The results of wgMLST used to determine the correlation between the three strains are shown in Fig. 4. Strain MFDS1004024 has 99.9% similarity with strain MFDS1006818, and the similarity between these two strains and strain MFDS1011653 was 99.6%, which contradicts the PFGE analysis. In conclusion, PFGE analysis showed that the two strains MFDS1004024 and MFDS1006818 had a high correlation and these two strains showed relatively low similarity (80.8%) with the strain MFDS1011653. In contrast, wgMLST results showed that the similarity of the three strains was ≥ 99.6%, indicating a very high similarity.

## Discussion

A multiple-school foodborne outbreak of *Salmonella* occurred in September 2018, which affected a total 2,975 patients, and this was recorded as the largest bacterial outbreak in Korea. The present study showed that the cause of this outbreak reported by approximately 60 school meal services was a single food source and originated from the same contaminated product of egg white liquid upon analyzing the characteristics of *S*. Thompson strains isolated from the investigation samples. We confirmed that *S*. Thompson strains with the same genotype were detected in the chocolate cake consumed by patients directly, chocolate cakes in distribution, whipper used by chocolate cake manufacturer, and egg white liquid which is one of the main ingredients of the cream applied on the chocolate cakes. Fifty-six strains harbored *stn* that causes diarrhea when *Salmonella* invade the intestines, *invA* which facilitates adhesion to and invasion into epithelial cells, *his* which is involved in regulating histidine transport, and *hin* which expresses a flagellum corresponding to two flagellar antigen phases 1 and 2, which are associated with pathogenicity. Generally, it has been reported that the primary process of salmonellosis involves the invasion of *Salmonella* into intestinal cells followed by proliferation. Salmonellosis is caused by the invasion of *Salmonella* into intestinal cells and fluid secretion, which causes inflammation of the lamina propria. Various virulence factors are involved in this process. Virulence factors are substances that are produced by pathogens and cause infections and diseases in the host. After exposure to pathogens, bacteria adhere to the skin or mucus membrane, and the adhered bacteria invade the host cell through the epithelium [19]. Genes in *Salmonella* associated with pathogenicity are involved in plasmid and chromosomal DNA replication, including *Salmonella* pathogenicity islands (SPIs). Upon invasion into the cells of an animal host, the type 3 secretion system encoded by SPIs is induced to secrete various virulence factors into the host cytoplasm to disrupt normal intracellular functions, leading to the promotion of invasion and proliferation of *Salmonella* in the cells. The *invA* gene is involved in this process and is an essential gene required for the adhesion and invasion of pathogenic strains that cause food poisoning. It has a *Salmonella*-specific sequence that is used to detect *Salmonella* in various samples [20, 21]. The present study found a trend similar to that reported in earlier studies in which *Salmonella* strains have been found to harbor *invA* [22, 23]. The *sefA* gene, which encodes thin filamentous fimbria of *S*. Enteritidis, was not detected in *S*. Thompson isolated in this study, and it has been reported to be observed specifically in serogroup D1 [18, 24]. In the case of *spv*, a gene that confers pathogenicity derived from a plasmid that can specifically detect *S*. Enteritidis, studies conducted by Araque and Chaudhary *et al*. [25, 26] reported that the gene has not been detected in any *S*. Thompson isolates. The *invA*, *stn*, and *hin* genes were detected in the *Salmonella* isolates analyzed in this study, indicating that they are closely related to the occurrence of food poisoning. According to Kim *et al*. [27], *Salmonella* strains with monophasic flagella do not harbor the *hin* gene and all monophasic *Salmonella* strains show phase 1. In addition, the study reported that in the case of *Salmonella* bacteria that lack *hin*, the composition of O-antigens and phase 1 of H antigens can identify *Salmonella* serotypes without conducting a phase 2 test, as in this study. The *hin* gene is involved in *fliA*, *B*, and *C* regulation that express flagella of phases 1 and 2, and one type of flagella is occasionally generated owing to the loss of the *hin* gene. The toxin gene *stn* causes inflammation and facilitates intestinal invasion of *Salmonella* [28, 29] along with the regulation by Hin recombinase expressed by the *hin* gene in *Salmonella* chromosome.

The PFGE pattern was identified to be the same for the 56 strains, demonstrating that the foodborne outbreaks that occurred in ten different areas were caused by the same strains and sources. Additional wgMLST analysis revealed that the isolated strains were 100% identical, with small differences in the number of alleles by 2 to 3 alleles. The analysis led to the conclusion that outbreaks were caused by consuming chocolate cake, a single source, and that the egg white liquid used for preparing the cream of the chocolate cake was contaminated with the same *S*. Thompson strain.

The occurrence of global outbreaks associated with *S*. Thompson have been linked to smoked salmon (Netherlands in 2012), chicken (Shawarma; Canada in 2016), cilantro (California, USA in 2001), and contamination of beef and bread [30-34]. Of the 403 food poisoning cases caused by *Salmonella* for 10 years from 1998 to 2008, six cases were caused by *S*. Thompson, accounting for 1.5% of the total cases [35]. The most common *Salmonella* serotypes detected in foodborne outbreaks in Korea over the past five years are S. Enteritis, S. Heidelberg, and S. Infantis, and there have been only two cases of food poisoning caused by *S*. Thompson in Korea until this outbreak occurred in 2018. In relation to the *S*. Thompson food poisoning incident compared to that in the past, one of the two strains isolated was a strain previously isolated from a crab stick in 2014 (MFDS1004024), an ingredient of gimbap left at a restaurant in Hwasun-gun, Jeonnam-do after serving gimbap; 25 individuals who consumed gimbap at that restaurant had reported food poisoning symptoms. In addition, the strain isolated in 2015 (MFDS1006818) was detected in eggshells; eggs are used as an ingredient in rolled omelets served during breakfast and lunch at restaurants located in Jeonju-si, Jeonbuk-do. In total, 24 individuals who consumed the lunch box reported symptoms of food poisoning and were hospitalized. In cases of food poisoning at restaurants, investigations are often limited as the food does not remain at the time of the investigation, and additional examination of distribution is affected owing to missing distribution records or a narrow scope of food poisoning incidents. In particular, the outbreak that occurred in 2015 was found to be mainly caused by the fact that eggs, which are the main ingredients of rolled egg omelets, were stored at room temperature and that the rolled egg was cooked the night before and served the next day.

Based on the findings of the present study, the three strains isolated from the three outbreaks in Korea harbored the same genes and high genetic similarity. Since strains with the same genetic characteristics were detected continuously from 2014 to 2018, it may be possible that the land or water in the area was contaminated with *S*. Thompson or that the areas had the same source of contamination. This result can be considered as the basis for tracking and preventing contamination of the source related to *S*. Thompson. The investigation of an outbreak is complex and is limited based on human resources and time taken to trace the contamination route of origin in many cases of small-scale outbreaks. Therefore, tracking methods using advanced genetic technologies may represent an important alternative to overcome these limitations. If the contaminated food source and causative organisms are identified and independent outbreaks are found to be caused by similar strains, as is the case in this study, it can be used to determine the cause of contamination in the investigation process. Gene similarity can be used as a standard for identifying common features regarding the cause of independent outbreaks, distribution processes, and production stages, making it possible to identify the cause of outbreaks with limited manpower. In addition, if the contaminated food source has been discarded or is identified for independent outbreaks, and the characteristics and similarity of strains are matched, an analysis can be conducted to determine whether there are common contaminated foods or sources to identify the cause of food poisoning.

There are few reported cases of *S*. Thompson food poisoning worldwide, including Korea; however, this study identified the cause of *S*. Thompson foodborne outbreaks and analyzed the genetic characteristics of the strains. In addition, PFGE analysis showed that the three strains were not the same, while wgMLST analysis results indicated that the three strains had a high level of similarity. This result was supported by the finding that single nucleotide polymorphism (SNP) differences among the three strains were less than 60 SNPs (data not shown). In conclusion, wgMLST analysis was able to provide highly resolved phylogeny and the combination of characterization methods of target gene PCR and PFGE with wgMLST analyses can be effective in providing evidence during outbreak investigation.

## Figures and Tables

**Fig. 2:**
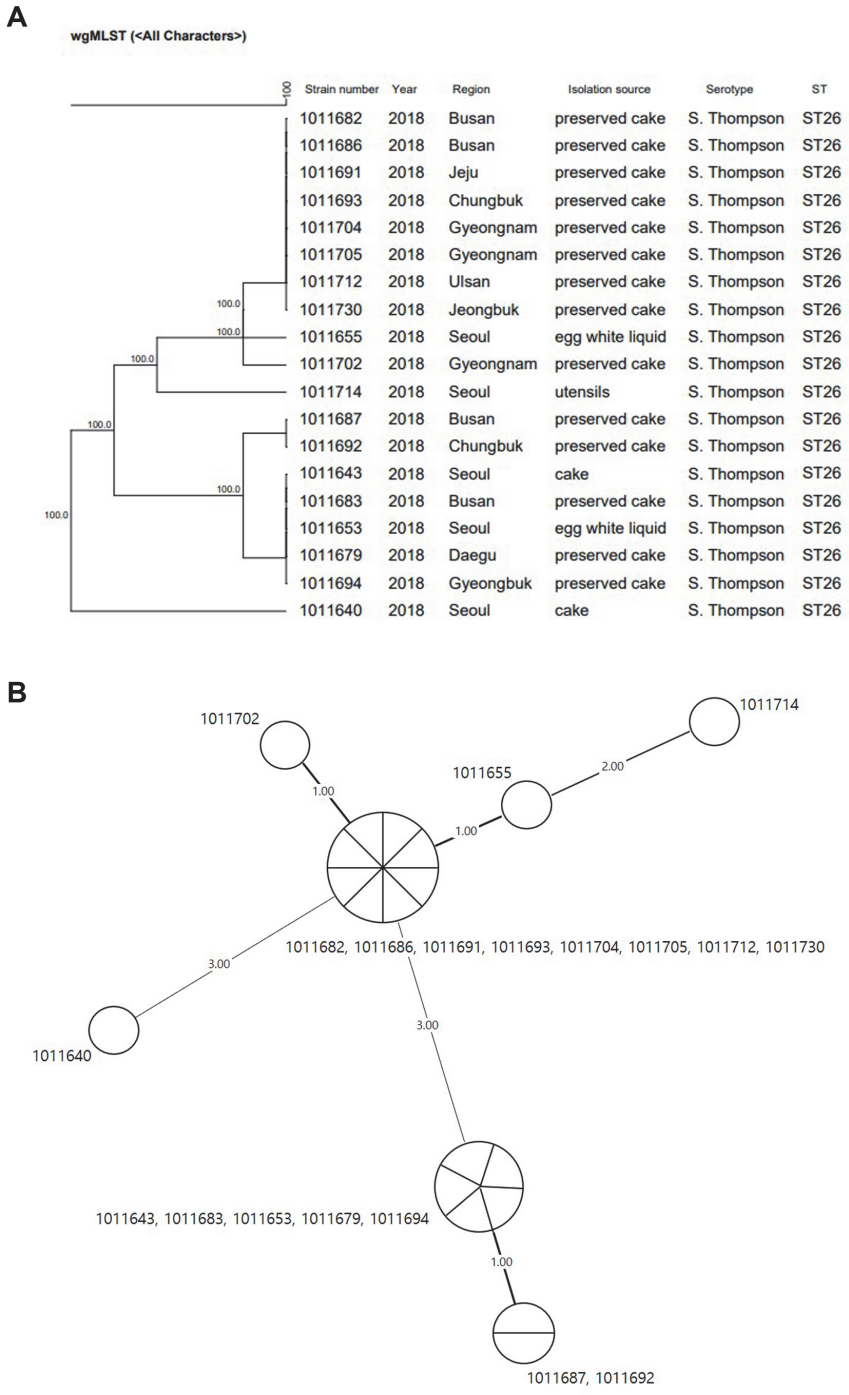
Phylogenetic tree of *Salmonella* Thompson strains: A) wgMLST results show the evolutionary distance of the 19 strains of isolates which were confirmed to be 100% identical. B) Minimum spanning tree of 19 strains shows the allelic distance between each strain that is closely correlated; the seven digits indicate strain number and the number between the circle shows the allelic distance. MST was constructed in BioNumerics 7.6 using wgMLST data for isolates sequenced in this study. wgMLST, whole-genome multi-locus sequence typing.

**Table 1:** Primers/probes and PCR conditions used in the present study.

Target gene	Sequence (5′-3′)	Size (bp)	PCR cycling conditions
*spvC*	F: AATGAACTACGAAGTGGGCG	112	50°C, 2 min → 95°C, 10 min → 95°C,
	R: TCAAACGATAAAACGGTTCCTC		15 s → 60°C, 1 min: 40 cycles
	P: FAM-ATGGTGGCGAAATGCAGAGACAGGC-BHQ1		
*sefA*	F: GGCTTCGGTATCTGGTGGTGTA	98	50°C, 2 min → 95°C, 10 min → 95°C,
	R: GGTCATTAATATTGGCCCTGAATA		15 s → 60°C, 1 min: 40 cycles
	P:Cy5-CCACTGTCCCGTTCGTTGATGGACA-BHQ2		
*hin*	F: TCCATGAGAAAAGCGACTAAAAT	572	95 °C, 3 min → 95 °C, 30 s → 57 °C, 30 s →
	R: AGCCGACTAATCTGTTCCTGTTC		72°C, 1 min: 30 cycles → 72°C, 2 min

PCR, Polymerase Chain Reaction.

**Table 2:** Food and environmental isolates analyzed in the present study.

No.	Isolate	Strain No. of MFDS	Region	Sample type
1	*S.* Thompson	1011630	Kwangju	Cake (chocolate)
2	*S.* Thompson	1011640	Seoul	Cake (strawberry)
3	*S.* Thompson	1011643	Seoul	Cake (white)
4	*S.* Thompson	1011653	Seoul	Egg White Liquid
5	*S.* Thompson	1011655	Seoul	Egg White Liquid
6	*S.* Thompson	1011678	Daegu	Cake (Preserved in elementary school)
7	*S.* Thompson	1011679	Daegu	Cake (Preserved in kindergarten)
8	*S.* Thompson	1011680	Daegu	Cake (Preserved in elementary school)
9	*S.* Thompson	1011681	Daegu	Cake (Preserved in middle school)
10	*S.* Thompson	1011682	Busan	Cake (Preserved in middle school)
11	*S.* Thompson	1011683	Busan	Cake (Preserved in middle school)
12	*S.* Thompson	1011684	Busan	Cake (Preserved in middle school)
13	*S.* Thompson	1011685	Busan	Cake (Preserved in elementary school)
14	*S.* Thompson	1011686	Busan	Cake (Preserved in high school)
15	*S.* Thompson	1011687	Busan	Cake (Preserved in high school)
16	*S.* Thompson	1011688	Busan	Cake (Preserved in high school)
17	*S.* Thompson	1011689	Busan	Cake (Preserved in middle school)
18	*S.* Thompson	1011690	Busan	Cake (Preserved in elementary school)
19	*S.* Thompson	1011691	Jeju	Cake (Preserved in elementary school)
20	*S.* Thompson	1011692	Chungbuk	Cake (Preserved in middle school)
21	*S.* Thompson	1011693	Chungbuk	Cake (Preserved in high school)
22	*S.* Thompson	1011694	Gyeongbuk	Cake (Preserved in elementary school)
23	*S.* Thompson	1011695	Gyeongbuk	Cake (Preserved in high school)
24	*S.* Thompson	1011696	Gyeongbuk	Cake (Preserved in elementary school)
25	*S.* Thompson	1011697	Gyeongbuk	Cake (Preserved in high school)
26	*S.* Thompson	1011698	Gyeongbuk	Cake (Preserved in middle school)
27	*S.* Thompson	1011699	Gyeongbuk	Cake (Preserved in middle school)
28	*S.* Thompson	1011700	Gyeongnam	Cake (Preserved in high school)
29	*S.* Thompson	1011701	Gyeongnam	Cake (Preserved in high school)
30	*S.* Thompson	1011702	Gyeongnam	Cake (Preserved in (High school)
31	*S.* Thompson	1011703	Gyeongnam	Cake ( Preserved in (High school)
32	*S.* Thompson	1011704	Gyeongnam	Cake (Preserved in Middle school)
33	*S.* Thompson	1011705	Gyeongnam	Cake (Preserved in High school)
34	*S.* Thompson	1011706	Gyeongnam	Cake (Preserved in Elementary school)
35	*S.* Thompson	1011707	Gyeongnam	Cake (Preserved in Middle school)
36	*S.* Thompson	1011708	Gyeongnam	Cake (Preserved in Middle school)
37	*S.* Thompson	1011709	Gyeongnam	Cake (Preserved in Middle school)
38	*S.* Thompson	1011710	Gyeongnam	Cake (Preserved in Elementary school)
39	*S.* Thompson	1011711	Gyeongnam	Cake (Preserved in high school)
40	*S.* Thompson	1011712	Ulsan	Cake (Preserved in middle school)
41	*S.* Thompson	1011713	Ulsan	Cake (Preserved in high school)
42	*S.* Thompson	1011714	Seoul	Whipper
43	*S.* Thompson	1011718	Jeonbuk	Cake (Preserved in elementary school)
44	*S.* Thompson	1011719	Jeonbuk	Cake (Preserved in middle school)
45	*S.* Thompson	1011720	Jeonbuk	Cake (Preserved in middle school)
46	*S.* Thompson	1011721	Jeonbuk	Cake (Preserved in elementary school)
47	*S.* Thompson	1011722	Jeonbuk	Cake (Preserved in elementary school)
48	*S.* Thompson	1011723	Jeonbuk	Cake (Preserved in elementary school)
49	*S.* Thompson	1011724	Jeonbuk	Cake (Preserved in high school)
50	*S.* Thompson	1011725	Jeonbuk	Cake (Preserved in elementary school)
51	*S.* Thompson	1011726	Jeonbuk	Cake (Preserved in elementary school)
52	*S.* Thompson	1011727	Jeonbuk	Cake (Preserved in elementary school)
53	*S.* Thompson	1011728	Jeonbuk	Cake (Preserved in elementary school)
54	*S.* Thompson	1011729	Jeonbuk	Cake (Preserved in middle school)
55	*S.* Thompson	1011730	Jeonbuk	Cake (Preserved in elementary school)
56	*S.* Thompson	1011731	Jeonbuk	Cake (Preserved in elementary school)

MFDS, Ministry of Food and Drug Safety, Rep. of Korea.

**Table 3:** PCR and serological typing result of *Salmonella* Thompson.

Strain	Serovar	Serological type			Real-time PCR					PCR

		O-antigen group	H Phase 1	H Phase 2	*his*	*invA*	*stn*	*sefA*	*spvC*	*hin*
56 isolates	*S*. Thompson	C	k	1, 5	+	+	+	-	-	+

PCR, Polymerase Chain Reaction.

**Table 4:** Strains isolated from *Salmonella* Thompson-related foodborne outbreaks in Korea and their characteristics.

Collection Date	Source	Point-of-Service	Serotype	Genes present	Gene Bank No.	Genetic Info.
5-Sep -2014	Crab-stick	Restaurant	*S*. Thompson O:7, H(k:1,5)	*his* (+), *invA* (+) *hin* (+) *stn* (+)	MFDS1004024	ST-26
29-June-2015	Egg (Shell)	Restaurant	*S*. Thompson O:7, H(k:1,5)	*his* (+), *invA* (+) *hin* (+) *stn* (+)	MFDS1006818	ST-26 SP6X01.009
9-Sep-2018	Egg White Liquid	School	*S*. Thompson O:7, H(k:1,5)	*his* (+), *invA* (+) *hin* (+) *stn* (+)	MFDS1011653	ST-26 SP6X01.011
